# SmShb, the SH2-Containing Adaptor Protein B of *Schistosoma mansoni* Regulates Venus Kinase Receptor Signaling Pathways

**DOI:** 10.1371/journal.pone.0163283

**Published:** 2016-09-16

**Authors:** Marion Morel, Mathieu Vanderstraete, Katia Cailliau, Steffen Hahnel, Christoph G. Grevelding, Colette Dissous

**Affiliations:** 1 Univ. Lille, CNRS, Inserm, CHU Lille, Institut Pasteur de Lille, U1019 –UMR 8204—CIIL—Center for Infection and Immunity of Lille, Lille, France; 2 Univ. Lille, CNRS, UMR 8576—UGSF—Unité de Glycobiologie Structurale et Fonctionnelle, Lille, France; 3 BFS, Institute for Parasitology, Justus-Liebig-University, Giessen, Germany; Hungarian Academy of Sciences, HUNGARY

## Abstract

Venus kinase receptors (VKRs) are invertebrate receptor tyrosine kinases (RTKs) formed by an extracellular Venus Fly Trap (VFT) ligand binding domain associated via a transmembrane domain with an intracellular tyrosine kinase (TK) domain. *Schistosoma mansoni* VKRs, SmVKR1 and SmVKR2, are both implicated in reproductive activities of the parasite. In this work, we show that the SH2 domain-containing protein SmShb is a partner of the phosphorylated form of SmVKR1. Expression of these proteins in *Xenopus* oocytes allowed us to demonstrate that the SH2 domain of SmShb interacts with the phosphotyrosine residue (pY979) located in the juxtamembrane region of SmVKR1. This interaction leads to phosphorylation of SmShb on tyrosines and promotes SmVKR1 signaling towards the JNK pathway. SmShb transcripts are expressed in all parasite stages and they were found in ovary and testes of adult worms, suggesting a possible colocalization of SmShb and SmVKR1 proteins. Silencing of SmShb in adult *S*. *mansoni* resulted in an accumulation of mature sperm in testes, indicating a possible role of SmShb in gametogenesis.

## Introduction

Schistosomiasis is the second most important parasitic disease in the world with more than 240 million people infected in tropical and subtropical areas and is responsible for about 200 000 deaths per year [1]. Praziquantel (PZQ) is the drug used currently to cure schistosomiasis. It is safe, affordable and effective against the three major species of schistosomes infecting humans (*Schistosoma mansoni*, *S*. *haematobium and S*. *japonicum*) but its massive administration in endemic areas has increased the likelihood of emergence of PZQ-tolerant parasites [2–5]. Therefore, there is a necessity to find new drugs and targets against schistosomes, and inhibiting parasite reproduction could be an excellent strategy to control both transmission and pathology of schistosomiasis. Indeed, the pathology mainly results from the impressive fertility of female worms which lay hundreds of eggs every day. Trapping of parasite eggs in host tissues finally causes severe disorders, particularly hepatosplenomegaly, hepatic fibrosis, and bladder cancer [6].

Protein kinases, and particularly tyrosine kinases (TKs), play important roles in development and metabolism. Receptor tyrosine kinase (RTK) signaling networks are essential for many cellular activities such as growth, proliferation, differentiation or migration, and they represent major targets in anti-cancer therapy [7]. Different studies have demonstrated the importance of TKs in *S*. *mansoni* gametogenesis and egg production indicating that parasite kinases are interesting targets for the development of new therapeutics against schistosomes [8–10]. Particularly, we have recently demonstrated the importance of Venus Kinase Receptors (VKRs) in the control of reproduction in *S*. *mansoni* [11]. VKRs are atypical RTKs formed by an extracellular Venus Fly Trap (VFT) domain associated via a single transmembrane domain with an intracellular TK domain similar to that of insulin receptors (IR) [12,13]. Since their discovery in *S*. *mansoni* in 2003 [12], VKRs have been identified in diverse invertebrate phyla including platyhelminths, arthropods, annelids, mollusks, echinoderms, and cnidarians [13,14]. Their function in reproduction has been confirmed in the mosquito *Aedes aegypti* recently [15]. Most of the organisms studied express a single VKR but in several species, multiple vkr genes are present and encode different VKR homologs [14]. Two VKR molecules, SmVKR1 and SmVKR2, are expressed in *S*. *mansoni*, and they were shown to participate in the development of the ovary in female worms. SmVKR1 and SmVKR2 are two different receptors that are activated by distinct ligands [16]. First evidence was obtained that these schistosome VKRs can differentially induce kinase signaling pathways. When they were expressed in *Xenopus* oocytes, each receptor activated both ERK2 and Akt pathways but only SmVKR1 was able to induce JNK phosphorylation [11,17].

Src Homology-2 (SH2) domain-containing protein B (Shb) proteins are pleiotropic adaptor proteins that convey signals from membrane RTKs to intracellular signaling intermediates. They possess a C-terminal SH2 domain and a central PhosphoTyrosine Binding (PTB) domain both involved in interaction with phosphorylated tyrosines, as well as an N-terminal Proline-rich sequence that binds SH3 domains [18]. Shb proteins enable protein-protein interactions and serve as platforms linking activated RTKs to various downstream signaling partners [19,20].

In this paper, we show that a *S*. *mansoni* Shb protein (SmShb) interacts with the membrane receptor SmVKR1. This interaction is dependent on the phosphorylation of the juxtamembrane Y_979_ residue of SmVKR1, occurs through the SH2 domain of SmShb, and allows the activation of a specific JNK signaling pathway in *Xenopus* oocytes. *In situ* hybridization shows that SmShb transcripts localize as Smvkr1 transcripts [11] in the reproductive organs of the parasite and SmShb silencing experiments exhibit morphological effects within the testes suggesting a role of SmShb in sperm migration. All together, these data describe for the first time a molecular function of Shb proteins in JNK signaling and suggest the potential importance of SmShb in development and/or reproduction of *S*. *mansoni*.

## Materials and Methods

### Ethics statement

All experiments involving hamsters within this study have been performed in accordance with the European Convention for the Protection of Vertebrate Animals used for Experimental and other Scientific Purposes (ETS No 123; revised Appendix A) and have been approved by the committee for ethics in animal experimentation of the region Nord Pas de Calais France (authorization No. AF/2009) in the local animal house of the Pasteur Institute of Lille (Agreement No. A59-35009).

### Parasite material

A Puerto-Rican strain of *S*. *mansoni* is maintained by passage through albino *Biomphalaria glabrata* snails and *Mesocricetus auratus* golden hamsters. Adult schistosome pairs were collected by portal perfusion from infected hamsters at 42–45 days post infection.

### Molecular cloning of SmShb

Total RNA of adult schistosomes was isolated using the RiboPure™ RNA Purification Kit. cDNA was prepared using the GeneRacer™ Kit with the SuperScript™ III reverse transcriptase (Invitrogen) following the manufacturer’s instructions. 5’ and 3’ ends of SmShb were determined by RACE PCR using GeneRacer™ 5’, 3’, 5’ Nested and 3’ Nested primers associated with SmShb-specific primers (S1 Table). Amplified products were subcloned into pCR™4-TOPO® vector and sequenced (EurofinsDNA). The full-length (FL) SmShb sequence was then amplified using SH2protFLf/SH2protFLr primers (S1 Table) and cloned into a pCR™4-TOPO® vector. A second PCR was performed using SmShbFLREf/SmShbFLREr primers containing *ClaI* and *XhoI* restriction sites respectively and the obtained fragment was inserted into a pCR™4-TOPO® vector (SmShb-pCR™4-TOPO®).

### *In silico* analyses and phylogenetic studies

Sequences were analyzed using the LASERGENE package (DNAStar, Madison, WI, USA). BLASTp and BLASTn analyses of sequences obtained from the library screening were performed using the NCBI databank http://blast.ncbi.nlm.nih.gov/Blast.cgi. Shb, Grb2, Grb7, Nck and Shc protein sequences were aligned using ClustalW algorithm in the BioEdit v7.2.5 software, and manually corrected. Maximum likelihood tree was built using MEGA6 [21] under the JTT+G+I model, with 1000 bootstrap repetitions.

### Plasmid constructs

SmShb-pCR™4-TOPO® was cut by *ClaI* and *XhoI* and the SmShb sequence was inserted in frame into the pGADT7 AD expression vector (Clontech) using the T4 DNA Ligase (Invitrogen™) (SmShb-pGADT7). The SH2 domain, amplified from SmShb-pGADT7 using SH2domainf/SmShbFLREr primers containing respectively *EcoRI* and *XhoI* sites, was inserted in frame into the pGADT7 AD expression vector. Site-directed mutagenesis of constructs was performed using the Isis DNA polymerase™ (MP Biomedicals). The SmShb-pGADT7 construct was used as a template for the production of SmShb^ΔSH2^ mutant using as primers SmShbdelSH2f/SmShbdelSH2r in which G_3310_ is replaced by T to generate a stop codon and to create the SmShb^ΔSH2^ variant (AA sequence 1–1048). SmVKR1-pcDNA and SmVKR1 ICD YYRE-pGBKT7 plasmids (already described respectively in [16] and [22]) were used as templates to generate SmVKR1 Y_979_F mutants using SmVKR1Y979Ff/SmVKR1Y979Fr as primers. The SmVKR2 F_949_Y mutant was obtained similarly from the SmVKR2-pcDNA plasmid [16] using the SmVKR2F949Yf/SmVKR2F949Yr as primers.

### Direct Y2H interaction studies

Intracellular domains (ICD) of constitutively active SmVKRs (SmVKR1YYRE and SmVKR2YYRE) and of *S*. *mansoni* insulin receptors (SmIR1YYRE and SmIR2YYHE) inserted in fusion with the Gal4-BD of the pGBKT7 plasmid (described in [22]) were used to transform Y187 yeasts using the Lithium acetate method which were plated on selective growth media, as described in the Yeast Protocols Handbook (Clontech). SmShb-pGADT7 was used to transform AH109 cells. Mating between Y187 and AH109 cells was performed and diploid yeasts were selected on stringent quadruple dropout medium, SD-Leu/-Trp/-His/-Ade.

### Analysis of proteins expressed in *Xenopus laevis* oocytes

cRNAs encoding SmVKR and SmShb variant proteins were synthesized *in vitro* using the mMessage mMachine® T7 Kit (Ambion, USA) from pcDNA, pGBKT7 and pGADT7 plasmid constructs digested by *PmeI* (pcDNA) or by *HindIII* (pGBKT7 and pGADT7) restriction enzymes. cRNA solutions (1 mg/mL) were injected in stage VI *Xenopus laevis* oocytes according to the procedure previously described [23]. Kinase activation of full-length SmVKR receptors was obtained by the addition of external ligands (L-Arg, Ca^++^) in the incubation medium [16]. Germinal Vesicle BreakDown (GVBD) was detected after 15h by the appearance of a white spot at the centre of the animal pole. Immunoprecipitation of proteins was performed according to the procedures described previously [23]. Following 5 h of expression, oocytes were lysed in buffer A (50 mM Hepes pH 7.4, 500 mM NaCl, 0.05% SDS, 5 mM MgCl2, 1 mg ml−1 bovine serum albumin, 10 μg ml−1 leupeptin, 10 μg ml−1 aprotinin, 10 μg ml−1 soybean trypsin inhibitor, 10 μg ml−1 benzamidine, 1 mM PMSF, 1 mM sodium vanadate) and centrifuged at 4°C for 15 min at 10,000 g. Membrane pellets were resuspended and incubated for 15 min at 4°C in buffer A containing 1% Triton X-100 and then centrifuged under the same conditions. Membrane extracts were incubated with anti-V5, anti-Myc or anti-HA antibodies (1:100; Invitrogen) overnight at 4°C and immune complexes were precipitated on protein A-Sepharose beads and analyzed by Western blotting using anti-V5 (1:50,000), anti-Myc (1:50,000), anti-HA (1:50,000) or PY20 (1:10,000; anti-phosphotyrosine, BD Biosciences) antibodies and the advanced ECL detection system (Amersham Biosciences) as previously described [11]. Immunoprecipitation of SmVKR ICD variants was performed from soluble extracts of oocytes lysed in buffer A containing 0.5% Triton X-100 and centrifuged at 12,000 g for 15 min at 4°C, in the same conditions as above. In some experiments, soluble extracts were used to analyse the phosphorylation cascades induced by SmVKR in oocytes. Total cell lysates were analyzed by Western blotting using anti-ERK2, anti-Akt, anti-p70S6K, anti-JNK and antibodies against the phosphorylated forms of these proteins exactly as previously described [11].

### *In situ* hybridization

Adult worm pairs were fixed in Bouin's solution (picric acid/acetic acid/formaldehyde; 15/1/5) and embedded in paraplast (Paraplast plus, Sigma). Sections (5 μm) were incubated in xylol to remove paraplast. Following rehydration, slides were treated with proteinase K (1 μg/ml) and sections dehydrated. For hybridization, *in vitro* transcripts (corresponding to nt 616–1082 sequence of SmShb (Genbank Accession Number JN864885)) were synthesized and labeled with digoxigenin following the protocol of the manufacturer (Roche). The correct size of labeled sense and antisense transcripts was controlled by gel electrophoresis and the quality of RNA probes was checked by blotting and detection of digoxigenin using alkaline phosphatase-conjugated anti-digoxigenin antibodies, naphtol-AS-phosphatase, and Fast Red TR (Sigma). *In situ* hybridization was performed at 57°C for 16 h as previously described [24]. Sections were washed in 0.5×SSC (75 mM NaCl, 7.5 mM sodium citrate, pH 7) and detection of alkaline phosphatase on slides was achieved as described above.

### RNAi experiments

cDNA fragments of SmShb (610 bp) and Luciferase (357 bp, control) were generated by PCR using respectively the SmShb-pGADT7 plasmid or the pGL3-basic plasmid (Promega) as templates and SmShbRNAiT7f/ SmShbRNAiT7r and LucT7f/LucT7f as primers (S1 Table). dsRNA were prepared using the Megascript RNAi kit (Ambion) according to the manufacturer's instructions and quantified spectrophotometrically (NanoVue PlusTM, GE Healthcare). Eight worm couples were placed in 4mm cuvette with 100 μL of M199 medium containing 25 μg of dsRNA and electroporated using the square-wave protocol already described [25] with a single impulse of 125 V during 20 ms. Worms were then incubated in 2.5 mL of complete M199 medium (supplemented with 10% SVF, 10 mM HEPES pH 7.4, 60 μg/ml Rifampicin and 50 U penicillin/streptomycin) at 37°C under a 5% CO_2_ atmosphere. After 7 days, 5 worm couples were picked up for Confocal Laser Scanning Microscopy (CLSM) examination and knockdown of gene expression was monitored by quantitative RT-PCR in the remaining worms.

### Quantitative RT-PCR experiments

Total RNA was extracted from worm couples using TRIzol® reagent (Invitrogen) and reverse transcription was performed using the Superscript III RT-PCR System (Invitrogen). cDNA was used as template for PCR amplification using KAPA SYBR® FAST Universal 2× qPCR Master Mix kit (Clontech) and the ABI PRISM 7300 detection system (Applied Biosystems, Foster City, CA, USA). SmShbqPCRf/SmShbqPCRr primers (S1 Table) were used to amplify SmShb cDNA in triplicate assays. Tubulin cDNA (GenBank Accession Number M80214) was amplified with SmTubulinqPCRf/SmTubulinqPCRr primers and used as an internal control. For graphical representation, the delta-delta Ct (ΔΔCt) method was applied [26] to compare SmShb expression in dsSmShb interfered worms to control dsLuc treated parasites. Statistical significance of SmShb knockdown was evaluated using Student's t-test in the GraphPad Prism program (GraphPad Software Inc.).

### CLSM examination of parasites

Worms fixed in AFA (ethanol 95%, formalin 3% and glacial acetic acid 2%) were stained for 30 min in 2.5% Hydrochloric Carmin red (Certistain®, Merck), and then destained in acidic 70% ethanol. Following dehydration in 70%, 90% and 100% ethanol, worms were preserved as whole-mounts in Canada balsam (Merck) on glass slides [27–29]. CLSM images were taken using a Leica LSM780 microscope with a 488 nm He/Ne laser and a 470 nm long-pass-filter under reflection mode.

## Results

### Identification and sequence analysis of SmShb

**Fig 1:**
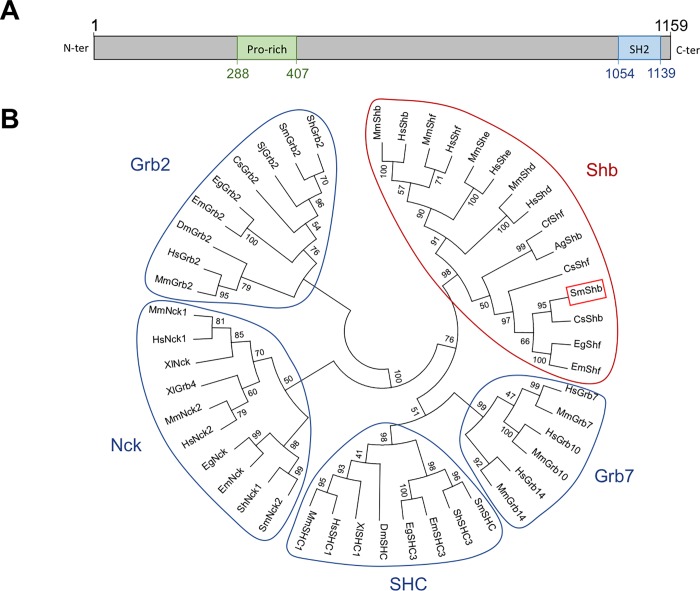
SmShb belongs to the Shb protein family. (A) SmShb is a protein of 1159 amino acids with a Proline-rich domain (AA288-407, in green) in its N-terminal part and an SH2 domain in its C-terminal end (AA1054-1139, in blue). (B) A phylogenetic tree was generated using the maximum likelihood method under the JTT+G+I model with a bootstrap test (1000 replicates). The tree was generated using the alignment of proteins from Shb (*Mus musculus* MmShb NP_001028478.1, MmShf NP_001013851.2, MmShe AAI09363.2, MmShd BAA33805.1; *Homo sapiens* HsShb CAA53091.1, HsShf Q7M4L6.2, HsShe Q5VZ18.1, HsShd Q96IW2.1; *Camponotus floridanus* CfShf EFN71155.1; *Anopheles gambiae* AgShb XP_309808.4; *Clonorchis sinensis* CsShf GAA56149.1, CsShb GAA50136.1; *Schistosoma mansoni* SmShb AFH41560.1; *Echinococcus granulosus* EgShf CDS15334.1 and *E*. *multilocularis* SmShf CDJ02881.1), Grb2 (*S*. *haematobium* ShGrb2 KGB37632.1; SmGrb2 CCD60977.1; *S*. *japonicum* SjGrb2 CAX75443.1; CsGrb2 GAA48411.1; EgGrb2 CDS18126.1; EmGrb2 CDJ01173.1; *Drosophila melanogaster* DmGrb2 NP_476858.1; HsGrb2 CAG46740.1 and MmGrb2 AAB40022.1), Grb7 (HsGrb7 BAA29059.1, HsGrb10 NP_005302.3, HsGrb14 AAC15861.1; MmGrb7 NP_034476.1, MmGrb10 AAB53687.1, MmGrb14 AAF43996.1), SHC (SmSHC CCD76305.1; ShSHC3 KGB39408.1; EmSHC3 CDJ05613.1; EgSHC3 EUB61901.1; DmSHC NP_524683.2; *Xenopus laevis* XlSHC1 NP_001083932.1; HsSHC1 NP_892113.4; MmSHC1 NP_001106802.1), and Nck (SmNck2 CCD77399.1; ShNck1 KGB32228.1; EmNck CDJ02918.1; EgNck CDS15372.1; HsNck2 NP_001004720.1, HsNck1 NP_001278928.1; MmNck2 NP_035009.3, MmNck1 NP_035008.2; XlGrb4 NP_001083313.1, XlNck AAH80058.1) families.

A partial sequence of a protein referred in Genbank databases as hypothetical SH2 domain-containing protein (Genbank Accession Number CCD77461) was shown from Y2H *S*. *mansoni* library screening to interact with the tyrosine-phosphorylated intracellular domain of SmVKR1 in its active form (SmVKR1 ICD YYRE) [11]. Using RACE-PCR, we have cloned and partially edited the full-length cDNA sequence of this interacting protein. It was shown to encode a member of the Shb adaptor protein family and named SmShb (Genbank Accession Number JN864885). BLAST analysis showed that the SmShb gene is present on chromosome 2 and is composed of 9 exons for a total length of about 113 Kb (S1 Fig). SmShb codes for a protein of 1159 residues containing a Pro-rich domain from positions 288 to 407 (exon 5) and a C-terminal SH2-domain from residues 1054 to 1139 encoded by exons 8 and 9 (Fig 1A). This sequence is similar to the SH2 domain-containing protein B (Shb) described for the first time by Welsh et al [18] with roles in signal transduction of ligand-activated RTKs [30]. The Shb protein family is composed of Shb together with the three paralogs Shd, She and Shf proteins [31,32], and is distinct from the SHC, Grb2, Grb7 or Nck families, which are all composed of adaptor proteins with SH2 domains together with other variable motifs for protein-protein interaction. Phylogenetic analyses indicated that SmShb belongs to the family of Shb proteins and is close to its ortholog CsShb from the trematode parasite *Clonorchis sinensis*. Furthermore, we showed that within the Shb family, platyhelminth (trematode and cestode) and insect proteins form two individual groups linked to the common branch of invertebrate Shb proteins, which is distinct from that of mammalian Shb proteins (Fig 1B).

### SmShb interacts with activated SmVKR1

**Fig 2:**
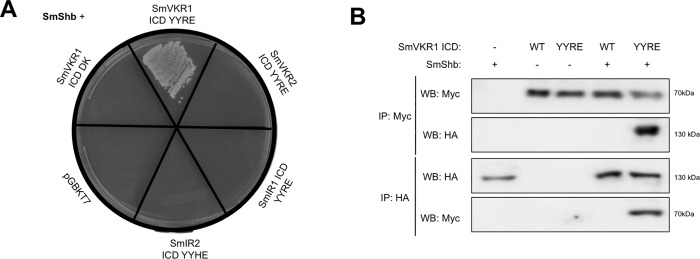
SmShb interacts specifically with activated SmVKR1. (A) Direct interaction in *S*. *cerevisiae* yeasts: AH109 yeasts expressing Gal4AD-fused SmShb were mated with Y187 yeasts expressing only Gal4DBD (pGBKT7), Gal4DBD-fused ICD YYxE (intracellular domain with active kinase) of SmVKR1, SmVKR2, SmIR1 or SmIR2, or Gal4DBD-fused ICD DK (dead kinase) of SmVKR1. Diploids were allowed to grow on a minimal SD -Leu/-Trp medium and diploids expressing interacting proteins were then selected on SD -Leu/-Trp/-His/-Ade medium. Only yeasts expressing SmShb and SmVKR1 ICD YYRE grew on the selective medium. (B) cRNAs encoding Myc-tagged SmVKR1 ICD WT or YYRE were co-injected in *Xenopus* oocytes with cRNA encoding HA-tagged SmShb. Oocytes were incubated for 5h in ND96 medium and lysed. Proteins from soluble extracts were immunoprecipitated (IP) by anti-Myc or anti-HA antibodies and immune complexes were analyzed by Western Blot (WB) to detect SmShb (130 kDa) and SmVKR1 ICD (68 kDa) with anti-HA or anti-Myc antibodies.

**Fig 3:**
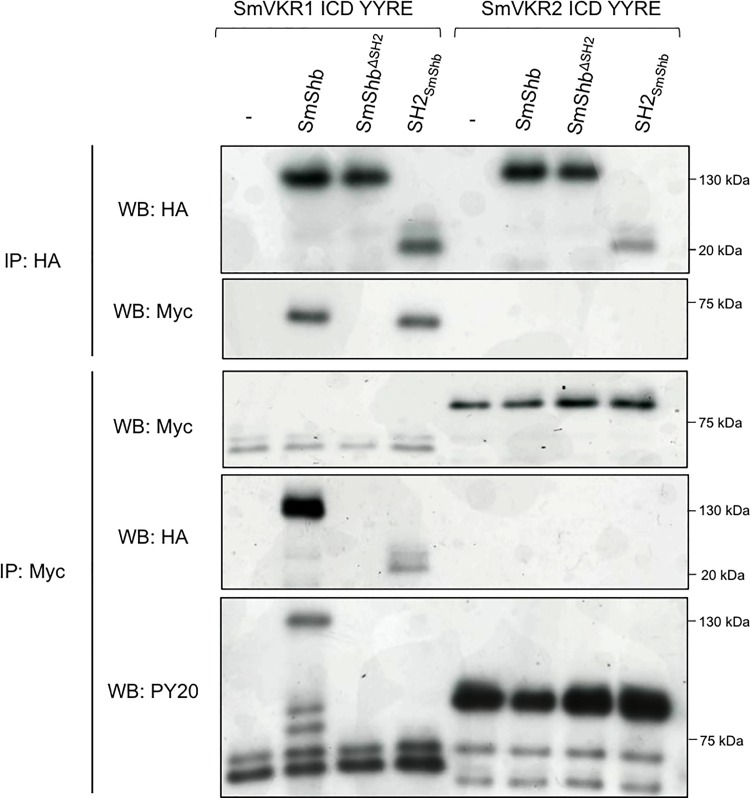
SmVKR1 interacts with SH2 domain and phosphorylates SmShb. cRNAs encoding Myc- SmVKR1 ICD YYRE or SmVKR2 ICD YYRE were injected in *Xenopus* oocytes with HA- SmShb, SmShb^ΔSH2^ (lacking the SH2 N-terminal domain) or the isolated domain SH2_SmShb_. Oocytes were incubated for 5h in ND96 medium and lysed. Proteins from soluble extracts were immunoprecipitated (IP) by anti-HA antibodies and analyzed by Western Blot (WB) with anti- HA and anti-Myc antibodies. HA-SmShb (130 kDa), HA-SmShb^ΔSH2^ (118 kDa) and HA-SH2_SmShb_ (10 kDa) were detected by anti-HA and Myc- SmVKR1 ICD YYRE (68 kDa) was detected in complexes made with HA-SmShb and HA-SH2_SmShb_ but not with HA-SmShb^ΔSH2^. Proteins from soluble extracts were also immunoprecipitated by anti-Myc antibodies and analyzed by Western Blot (WB) with anti- Myc, anti-HA and anti-phosphotyrosine (PY20) antibodies. Myc- SmVKR1 ICD YYRE (68 kDa) and SmVKR2 ICD YYRE (81 kDa) were detected and both HA-SmShb and HA-SH2_SmShb_ were detected in complexes made with Myc- SmVKR1 ICD YYRE. Anti-phosphotyrosine antibodies (PY20) confirmed the phosphorylation of SmVKR1 ICD YYRE and SmVKR2 ICD YYRE and revealed that of SmShb (130kDa) in the immune complexes formed with SmVKR1 ICD YYRE.

The full-length protein of SmShb was used to confirm its interaction with the kinase active intracellular domain of SmVKR1 (SmVKR1 ICD YYRE) in a direct binding assay in yeast. Similar interaction assays were also performed with the kinase active ICDs of SmVKR2 and of the two insulin receptors SmIR1 and SmIR2 of *S*. *mansoni* in order to study the specificity of the SmShb adaptor protein for the schistosome RTKs. Fig 2A shows that only diploid yeasts which expressed SmShb and SmVKR1 ICD YYRE could grow on the selective medium SD -Leu/-Trp/-His/-Ade suggesting an interaction between the two proteins. Moreover, the absence of growth for diploids expressing SmShb and the dead kinase (DK) version of SmVKR1 ICD strongly suggested that the interaction between SmShb and SmVKR1 was dependent on the phosphorylation of the receptor (Fig 2A). These results were confirmed by co-expressing these proteins in *Xenopus* oocytes (Fig 2B). Indeed, HA-tagged SmShb was co-immunoprecipitated with Myc-tagged SmVKR1 ICD YYRE (already shown to be tyrosine-phosphorylated in this system [22]), but not with the wild type and non-phosphorylated form of SmVKR1 ICD. In this system, no interaction was observed between the phosphorylated intracellular domain of SmVKR2 (SmVKR2 ICD YYRE) and SmShb (Fig 3), confirming that SmShb bound only to SmVKR1 in its phosphorylated form.

### The SH2 domain of SmShb binds to SmVKR1

The primary function of an SH2 domain is to recognize and to interact with phosphorylated tyrosines [33]. In order to assess the role of the SH2 domain of SmShb for its binding to activated SmVKR1, we produced two additional HA-tagged constructs of SmShb, one in which the SH2 domain was deleted (SmShb^ΔSH2^) and another one which encoded only the SH2 domain (SH2_SmShb_). Each construct (SmShb, SmShb^ΔSH2^, SH2_SmShb_) was co-expressed with SmVKR1 ICD YYRE in *Xenopus* oocytes and their ability to interact with SmVKR1 was analyzed by co-immunoprecipitation. The results indicated that SmVKR1 ICD YYRE co-precipitated with SmShb as well as with its SH2 domain but not with its SH2-deleted variant (Fig 3) and thus show that the SH2 domain was necessary and sufficient to permit SmShb interaction with the phosphorylated ICD of SmVKR1. Moreover, anti-phosphotyrosine (PY20) antibodies recognize a 130 kDa protein in SmShb-SmVKR1 ICD YYRE complexes. This protein was supposed to correspond to SmShb indicating that SmShb was probably a substrate of SmVKR1. In addition, SmVKR2 ICD YYRE, the second schistosome VKR, did not co-precipitate with any variant of SmShb, confirming the specificity of SmShb for SmVKR1 (Fig 3).

### SmShb recognizes the juxtamembrane residue Y_979_ of SmVKR1

**Fig 4:**
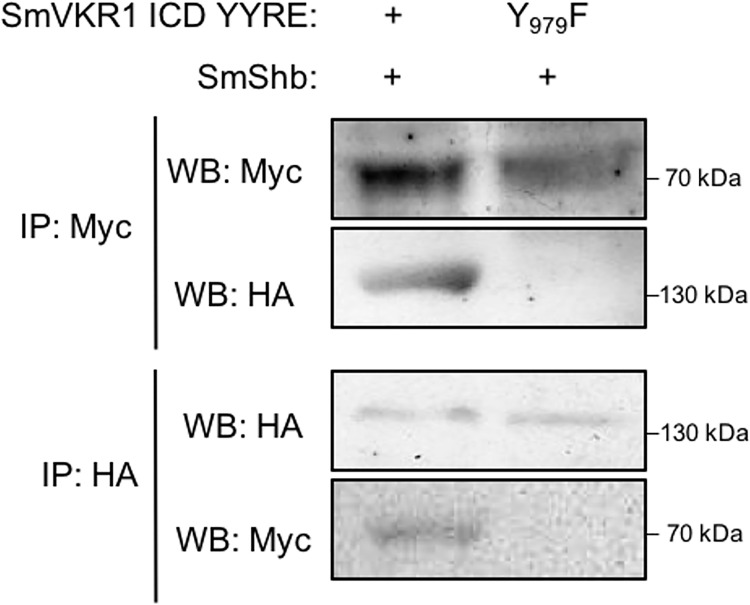
The juxtamembrane tyrosine (Y979) of SmVKR1 is required for SmShb binding. cRNAs encoding the intracellular domains (ICD) of Myc- SmVKR1 YYRE or SmVKR1 YYRE Y_979_F were co-injected in *Xenopus* oocytes with HA-SmShb and soluble extracts were immunoprecipitated (IP) by anti-Myc or anti-HA antibodies. Immune complexes were analyzed by Western Blot (WB) using anti-Myc or anti-HA antibodies. SmShb co-precipitated with SmVKR1 ICD YYRE but not with SmVKR1 ICD YYRE Y_979_F.

SH2 domains of Shb proteins have been shown to bind phosphorylated tyrosines (pY) included in the consensus motif pYxxL [34]. Within the SmVKR1 ICD sequence, we identified only one motif (Y_979_ENL) fitting to this consensus and found to be located within the juxtamembrane region of the receptor. In order to analyze the importance of this motif for the binding of SmShb to SmVKR1, we produced a mutant receptor (SmVKR1 Y_979_F) in which Y_979_ was replaced by a phenylalanine as a non-phosphorylable residue. We then compared the capacity of active ICD of SmVKR1 or SmVKR1 Y_979_F to co-precipitate with SmShb after co-expression in *Xenopus* oocytes. The results showed that SmShb was not able to interact with SmVKR1 Y_979_F, indicating that the residue Y_979_ was involved and required for the interaction of SmVKR1 ICD with the SH2 domain of SmShb (Fig 4).

**Fig 5:**
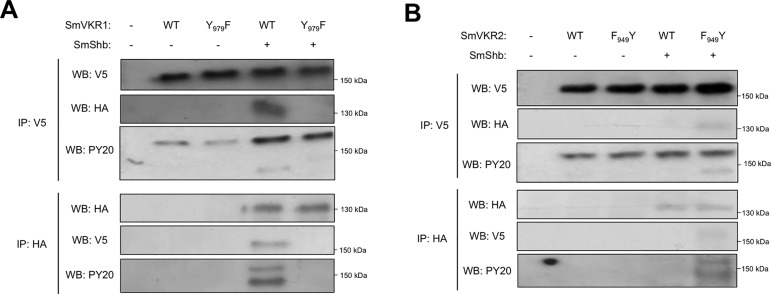
SmShb interacts with ligand-activated SmVKR1 and SmVKR2 receptors. (A) cRNA encoding V5-tagged SmVKR1 WT or SmVKR1 Y_979_F were injected with or without cRNA encoding HA-tagged SmShb in *Xenopus* oocytes. Following their expression, full-length receptors were activated by L-Arg to induce their auto-phosphorylation [11]. Proteins were then analysed by immunoprecipitation (IP) using anti-V5 or anti-HA antibodies followed by western blot (WB) using anti-V5, anti-HA or PY20 (anti-tyrosine phosphorylation) antibodies. Results demonstrated that only phosphorylated SmVKR1 WT bound to and phosphorylated SmShb as revealed by PY20 antibodies. (B) cRNA encoding V5-tagged SmVKR2 WT or SmVKR2 F_949_Y were injected with or without cRNA encoding HA-tagged SmShb in *Xenopus* oocytes. Following their expression, full-length receptors were activated by Ca^++^ to induce their auto-phosphorylation [11]. Proteins were then analysed by immunoprecipiration (IP) using anti-V5 or anti-HA antibodies followed by western blot (WB) analysis using anti-V5, anti-HA or PY20 (anti-tyrosine phosphorylation) antibodies. Results indicated that phosphorylated SmVKR2 WT did not bind SmShb whereas the mutated V5-SmVKR2 F_949_Y bound to and phosphorylated SmShb.

Since our aim was to further investigate the role of SmShb in the regulation of SmVKR1 activation pathways, it was necessary to confirm these previous observations in the context of a ligand-dependent activation of SmVKR1. According to this, full-length SmVKR1 WT and SmVKR1 Y_979_F proteins were co-expressed with SmShb in *Xenopus* oocytes and membrane receptors were further activated by L-Arginine (L-Arg) binding as previously described [16]. Western blot analysis using PY20 antibodies confirmed the kinase activity of RTKs which both auto-phosphorylate following binding of L-Arg. Results of co-immunoprecipitation also confirmed that the Y_979_ residue of SmVKR1 was required for its interaction with and for the phosphorylation of SmShb (Fig 5A).

Interestingly, the SmVKR2 receptor, which was not able to bind SmShb (Fig 3), contains an amino acid sequence F_949_EEL in its juxtamembrane part. To investigate its biological meaning with respect to binding of partners, we analysed the effect of the mutation of the F_949_EEL motif in a YxxL consensus (by a single F_949_Y exchange) on the SmShb binding potential of SmVKR2. Protein expression in oocytes and co-immunoprecipitation experiments were performed using the same conditions as described for SmVKR1, except that SmVKR2 receptors were activated by their specific ligands: Ca^++^ ions [16]. The results confirmed the data mentioned above showing that activated wild-type SmVKR2 did not interact with SmShb. However, activated SmVKR2 F_949_Y, which contains the pYxxL motif, interacted with SmShb, very likely by its SH2 domain (Fig 5B). Western blot results confirmed that SmShb effectively bound to phosphorylated SmVKR2 F_949_Y and is phosphorylated inside of this complex. Therefore, these data demonstrated that the juxtamembrane pYExL motif in SmVKR was necessary for SmShb interaction.

### SmShb regulates signaling by SmVKR1

**Fig 6:**
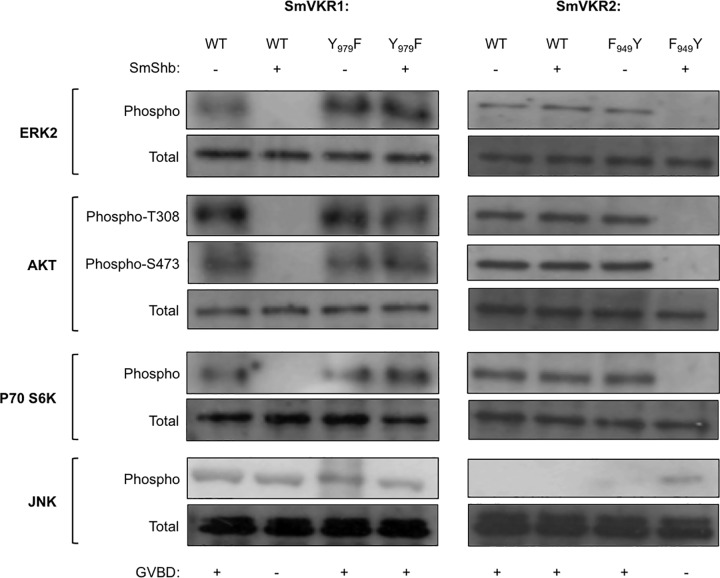
SmVKR-SmShb interaction induces JNK pathway. *Xenopus* oocytes were co-injected with cRNAs encoding V5-SmVKR1 WT or Y_979_F or V5-SmVKR2 WT or F_949_Y and SmShb and incubated with 1μM L-Arginine or 1mM Ca^++^ respectively. Oocyte lysates were analyzed by Western blot to detect phosphorylation of ERK2, Akt, S6K and JNK as described in Materials and Methods. In the absence of SmShb, SmVKR1 WT and Y_979_F activated ERK2, Akt, S6K and JNK pathways whereas SmVKR2 WT and F_949_Y activated ERK2, Akt, S6K but not JNK. However, in the presence of SmShb, SmVKR1 WT and SmVKR2 F_949_Y did no more activate ERK2, Akt, S6K. While JNK pathway was maintained with SmVKR1 WT, it was specifically induced by SmShb in the case of the SmVKR2 F_949_Y mutated and able to bind SmShb. In both cases, GVBD dependent on ERK2 and Akt activation was inhibited. SmShb did not affect signaling by SmVKR1 Y_979_F or SmVKR2 WT, which were not able to interact with SmShb.

**Fig 7:**
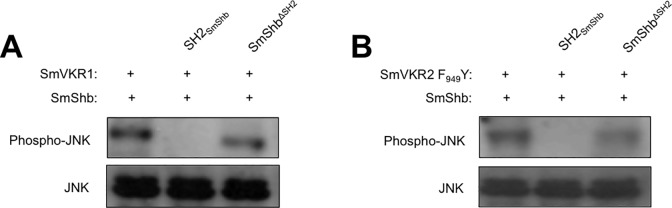
SmShb is a partner of the JNK pathway. cRNA (40 ng) of SmVKR1(A) or SmVKR2 F_949_Y (B) were injected in oocytes without or with cRNA (25 ng) encoding SH2_SmShb_ or SH2-deleted SmShb (SmShb^ΔSH2^). After two hours, a second injection of cRNA encoding SmShb (35 ng) was performed. Oocytes were further incubated for 3 hours then lysed. JNK phosphorylation was detected in oocyte lysates by western blotting using anti-phospho JNK antibodies. Pre-incubation of the SH2 domain with SmVKR1 and with SmVKR2 F_949_Y blocked the capacity of SmShb to induce JNK pathway.

Previous studies have shown that the activation of both SmVKR1 and SmVKR2 triggers ERK2 and PI3K/Akt/S6K pathways as well as meiosis resumption (or germinal vesicle breakdown (GVBD)) in *Xenopus* oocytes. Additionally, it was observed that activated SmVKR1, but not SmVKR2, induced the phosphorylation of JNK [11]. To analyze a possible regulatory function of SmShb on SmVKR1 signaling, the two proteins were co-expressed in *Xenopus* oocytes. Results (Fig 6) showed that in the presence of SmShb, ERK2 and PI3K/Akt/S6K pathways as well as GVBD induced by the activation of SmVKR1 were blocked in oocytes while the JNK pathway was still active. As expected, co-expression of SmShb with SmVKR1Y_979_F did not affect signaling pathways (Fig 6); this was in agreement with the absence of its binding to the mutated receptor (shown in Fig 5). These results suggest that SmShb could inhibit the activation properties of SmVKR1 in oocytes. However, the maintenance of JNK activation in oocytes expressing activated SmVKR1 and SmShb indicates that SmShb might be directly involved in JNK signaling processes. To test this hypothesis we made use of SmVKR2 F_949_Y, the SmVKR2 mutant that was able to interact with SmShb. Indeed, co-expression of SmShb with SmVKR2 F_949_Y induced the activation of a JNK pathway in oocytes which did not exist with SmVKR2 wild type and blocked ERK2 and PI3K/Akt/S6K pathways as well as GVBD (Fig 6). The specific role of SmShb in the induction of JNK activation by both SmVKR1 and SmVKR2 F_949_Y was supported by the demonstration that the activation of JNK in the presence of SmShb was blocked by an excess of SH2_SmShb_ protein that acts as a negative dominant of SmShb but not by an excess of SH2-deleted SmShb protein (SmShb^ΔSH2^) (Fig 7).

### Localization and functions of SmShb in adult schistosomes

**Fig 8:**
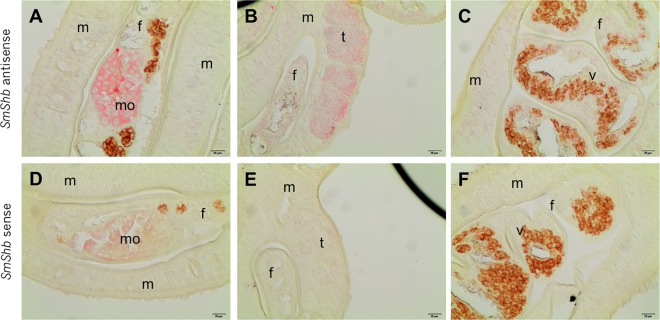
Localization of SmShb transcripts in sections of paired adult worms by *in situ* hybridization. SmShb transcripts were detected in mature oocytes (A), in testes (B) and lightly in the vitellarium (C). Sense probe of SmShb was used as control in D, E and F. (m: male; f: female; mo: mature oocytes; t: testes; v: vitellarium; scale bar = 20μm)

Previous studies have shown that Smvkr1 is expressed in various tissues of all parasite stages [12,16] and particularly in the reproductive organs of male and female worms, with a more intense labelling in the mature oocytes present at the posterior part of the ovary [11]. Here, *in situ* hybridization showed that SmShb transcripts were also detected in the mature part of the ovary (Fig 8A). Furthermore, SmShb transcripts were detected within the testicular lobes where signal intensity seemed to be more pronounced in their dorsal parts (Fig 8B). A faint labelling is also detected in other tissues, suggesting that SmShb is expressed ubiquitously in adult worms (Fig 8C). Recently performed RNAseq analyses confirmed the occurrence of SmShb transcripts in the gonads and further tissues of adult *S*. *mansoni* (Grevelding, unpublished). Moreover, as for Smvkr1 [16], RT-PCR experiments indicated that the SmShb gene is more actively expressed in larval stages than in adult worms (results not shown).

**Fig 9:**
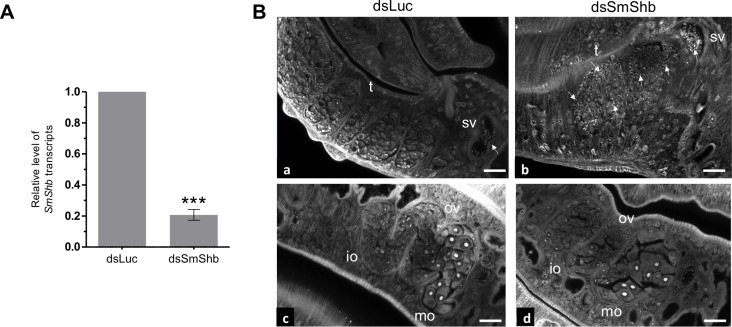
Effects of SmShb knock-down by RNA interference in adult worms. Worm couples were electroporated and incubated for 7 days either with dsSmShb or irrelevant dsLuc (control) as described in Materials and Methods. (A) Levels of SmShb transcripts were determined by quantitative RT-PCR in each worm sample. Interference led to a reduction of about 80% in the level of SmShb transcripts compared to controls. Statistical analysis was performed using the Student’s t-test and values are expressed as mean ± SEM of four determinations (*** p<0.001). (B) CLSM images of whole-mount preparations of male (a, b) or female (c, d) worms issued from worm couples electroporated with dsLuc (a, c) or dsSmShb (b, d). Accumulation of sperm in testicular lobes is observed in dsSmShb-treated males (b). Less mature oocytes were present in the posterior part of the female ovary (d). (ov: ovary; mo: mature oocytes; io: immature oocytes; t: testes; sv: seminal vesicle; arrows indicate mature sperm; scale bar = 20μm)

Attempts to analyse SmShb functions in schistosome biology have used its knockdown by RNA interference (RNAi) in adult worms. Seven days after electroporation with dsRNA, a decrease of about 80% SmShb transcripts was obtained, as compared to control worms electroporated with irrelevant dsRNA (dsLuc) (Fig 9A). SmShb silencing had no impact on worm viability, pairing or egg laying. In SmShb-interfered female worms, phenotypic analysis by CLSM showed discrete alterations of ovary structures with apparently a slight decrease of the number of cells. In contrast, in males, SmShb silencing had a remarkable effect on testes. Compared to the control, huge accumulation of differentiated sperm was found within the testicular lobes of male worms (Fig 9B).

## Discussion

Adaptor proteins are defined by their capacity to link membrane receptors to cytosolic proteins with enzymatic or transcriptional activation properties. Linking is mediated by diverse specific binding domains, like SH2 and PTB or SH3, which recognize phosphorylated tyrosines or poly-proline sequences, respectively. Shb is an adaptor protein containing an N-terminal Pro-rich domain, a central PTB domain and a C-terminal SH2 domain. The SH2 domain interacts with diverse phosphorylated RTKs preferentially at a pY-T/V/I-X-L binding site [30]. In contrast, the Pro-rich domain can recruit SH3-containing proteins like Src, p85 PI3K, PLC-γ, c-Abl, Janus Kinase (JAK), Grb2 or Eps8. Tyrosine phosphorylation of Shb in turn generates binding sites for other SH2-containing partners making Shb an essential platform to transduce various cellular signals [20].

Shb proteins are ubiquitously expressed and generate variable complexes depending on the cell type and on the conditions of cell activation. Therefore, they have unique effects either in differentiation, proliferation, survival or motility [19,20]. The Shb SH2 domain binds to PDGF-R[30], FGFR-1 [35], VEGFR-2 [36], NGF-R [37] to regulate apoptosis, proliferation, cell migration, and differentiation respectively in fibroblasts, endothelial, and neuronal cells through the modulation of Ras/MEK/MAPK and PI3K kinase pathways. Shb can modulate c-Abl pro-apoptotic signaling in response to stress stimuli by interacting with SH2 and SH3 domains of the Abl kinase and regulating its activity [38]. In mouse embryonic stem cells, the absence of Shb impairs various aspects of differentiation, suggesting a role of Shb in development [39,40]. Moreover, transgenic mice deficient in the Shb gene exhibit impairment of glucose homeostasis due to a decrease of insulin secretion [20,41], hematopoietic disorders [42,43] and abnormalities in reproduction linked to increased ERK/S6K signaling in ovary cells [44].

While much is known about the various physiological roles of Shb in mammals [20], the functions of Shb orthologs in invertebrates have not been studied yet. This paper presents i) the first characterization of an invertebrate Shb protein expressed by a platyhelminth parasite, ii) its interaction with an unusual RTK, VKR, which specifically occurs in invertebrates, iii) the demonstration for the first time of the potential participation of Shb to JNK activation, and iv) the hypothesis that this pathway could be involved in gamete mobility and thus in reproduction.

The protein SmShb has a structure similar to other proteins of the Shb family with the exception of the PTB domain, a sequence demonstrated to interact with the FAK focal adhesion kinase [19], which was not identified in SmShb. Phylogenetic analyses however, confirm that SmShb belongs to the Shb family and is distinct from Shc, Grb2, Grb7 or Nck proteins which represent other SH2-containing adaptors largely recognized for their capacity to bind to activated RTKs and for their involvement in signaling.

Since their first discovery in 2003 [12], VKRs have been identified in various organisms and shown to be expressed in larval stages and gonads of several animal species [12–14]. Recent advances demonstrated their importance for reproduction in the *S*. *mansoni* parasite and in the insect *A*. *aegypti* [45] but their functions in signaling remain elusive. In this work, we identified the protein SmShb as an interacting partner of SmVKR1.

Indeed, analyses of the capacity of SmShb to interact with various known RTKs of *S*. *mansoni*, ie SmVKR1, SmVKR2, SmIR1 and SmIR2, expressed either in their normal or in their active kinase states, have shown that the intracellular domain of the constitutively active SmVKR1 kinase was the only one to offer binding sites for SmShb. Identical results were obtained from interaction studies in yeast and in *Xenopus* oocytes. We also provided evidence that the specific binding of SmShb to the phosphorylated form of SmVKR1 was mediated by its SH2 domain and through the unique motif pY_979_ENL located in the intracellular part of SmVKR1, just next to the transmembrane domain. Mapping of the phosphorylable tyrosines present within the SmVKR1 intracellular sequence effectively indicated that only the residue Y_979_ was susceptible to be part of the consensus motif pYxxL for Shb SH2 domain binding [30]. Mutagenesis experiments then allowed us to confirm that Y_979_ was a crucial residue for the interaction between phosphorylated SmVKR1 and SmShb. Moreover, in the case of SmVKR2 which does not bind SmShb (and possesses a juxtamembrane F_949_EEL in place of the Y_979_ENL of SmVKR1), the replacement of F_949_ by a tyrosine residue was sufficient to allow its interaction with SmShb. These data indicated that a juxtamembrane motif pYExL in SmVKR was necessary and sufficient to allow interaction with SmShb. SmIR1 and SmIR2 insulin receptors, which do not possess similar motifs in their juxtamembrane regions, did not bind SmShb. However, it is important to mention that, in the experimental conditions used for SmVKR receptors, the activated *Schistosoma* EGF receptor (SER), which contains a juxtamembrane YxxL (YTNL) motif similarly to that of SmVKR1, did not bind SmShb (results not shown). This suggests that the Y+1 residue E could be required for SH2_SmShb_ binding. Additionally, it is possible that the interaction between SmShb and SmVKRs requires the participation of specific residues surrounding the phosphorylated tyrosine, such as proline at Y-1 and cysteine at Y+4 positions. Indeed, these residues are common to SmVKR1 (PpYENLC) and mutated F_949_Y SmVKR2 (PpYEELC) but they are not present in SER (ApYTNLL). Further experiments are still necessary to fully define the binding site of SmShb on SmVKRs, and to check for possible interaction of this adaptor protein with several other RTKs already characterized in *S*. *mansoni*, particularly with the parasite FGF-R receptors [46]. As mammal Shb proteins have been shown to interact with a large variety of RTKs [20], we cannot exclude that SmShb interacts as well with other schistosome RTKs. Furthermore, this result indicates that changing the pYExL motif to FEEL allows discrimination of SmVKRs by their potential downstream interaction partners substantiating the view that different and non-redundant roles of the schistosome VKRs have evolved after the duplication event of the first schistosome VKR gene. More largely, a possible species-restriction of the binding between Shb and VKR proteins could exist since we have observed that AgVKR from the mosquito *Anopheles gambiae* was not able to interact with SmShb while inversely the SH2 domain of AgShb (XP_309808.4), which shares about 40% homology with SH2_SmShb_, interacted with AgVKR but not with SmVKR1 (results not shown).

In a previous study we have demonstrated that the activation of SmVKR1 and SmVKR2 induced ERK2 and PI3K/Akt/S6K pathways in *Xenopus* oocytes and that only SmVKR1 activated the phosphorylation of JNK [11]. In this work, we showed that in *Xenopus* oocytes ligand-activation of SmVKR1 in the presence of SmShb resulted in JNK phosphorylation, but at the same time in a blockade of ERK and PI3K pathways and as a consequence in the inhibition of meiosis resumption in the oocyte. These results already suggested that SmShb could be involved in the initiation of a JNK cascade activation and further experiments performed with the SmVKR2 receptor mutated to be able to bind SmShb then confirmed the potential role of SmShb in the induction of JNK activation. Ligand activation of SmVKR2 F_949_Y in oocytes in the presence of SmShb generated a phenotype similar to that of ligand-activated SmVKR1, with a stimulation of the JNK pathway concomitant to the inhibition of ERK and PI3K pathways and GVBD. To our knowledge, the participation of Shb proteins in the activation of JNK has not been shown in mammalian cells, but their function in the regulation of ERK and Akt signaling has been described in Shb-deficient mice [20]. The loss of Shb provoked an increase of ERK or Akt signaling in different cells, suggesting that Shb could facilitate cell differentiation by negatively modulating ERK and Akt activation. Consistently, our results confirm here the function of SmShb in the inhibition of ERK and Akt signalling which are two predominant VKR pathways.

SmVKR1 and SmShb were expressed in all parasite stages with a more important quantity of transcripts detected in larval stages as compared to adult parasites. However, in sexually mature *S*. *mansoni*, both types of transcripts were co-localized in reproductive organs (mature oocytes and testes), suggesting that SmVKR1 and Shb proteins could interact within gametes. However, SmShb silencing in adult worm pairs did not provide solid morphological arguments for a decisive role of SmVKR1-SmShb interaction in the ovary. Knocking-down of SmVKR1 had shown an accumulation of big oocytes [11], which has not been as obvious in the ovaries of SmShb-silenced females. However, SmShb knock-down in male schistosomes led to an accumulation of differentiated gametes in testes, a phenotype which was not observed before in SmVKR1-silenced males [11]. As many other RTKs, VKRs probably exert pleiotropic functions and induce the activation of various signaling pathways controlling metabolism, growth and differentiation. Moreover, Shb proteins, whose expression has been shown to be tightly controlled in cells [20], are multifaceted signaling partners for diverse RTKs and their platform-based activities are strictly dependent on the cellular context including receptor activation and tissue- and stage-dependent expression of potential binding partners. This is supported by our recent studies describing many proteins as potential partners of SmShb and whose identities revealed their functions in various biological processes. This in turn explains that the congruence of Smvkr1 and SmShb RNAi phenotypes was small. Nonetheless, localization and RNAi results provided a first view on SmShb function, which is associated with gametogenesis.

In conclusion, SmShb appears to be a component of a signal transduction platform specifically formed by activated SmVKR1. In this molecular context activated SmShb has the potential to mediate the activation of a JNK pathway and negatively modulate ERK- and Akt-dependent metabolism and proliferation pathways, supporting cell differentiation and development pathways. SmShb and SmVKR1 are expressed in all the stages of the parasite and they are particularly abundant in larval forms. It would be interesting to unravel the importance of their partnership in the development of other *S*. *mansoni* stages such as schistosomula and sporocysts.

## Supporting Information

S1 Fig Gene organization of SmShb. (A) Exon-intron structure of SmShb locus. Exons are shown as blue boxes and introns as green lines. Size scale is identical for introns and exons, except for I1 and I2 (larger than 30kb). (B) Schematic representation of the SmShb transcript indicating the coding sequence (in grey) with the Proline-rich domain in green and the SH2 domain in light blue. Untranslated regions (5’- and 3’-UTR) are indicated in orange.
(TIF)

S1 Table List of oligonucleotide sequences used as primers. (PDF)
