# Supplementary figures and images for: SmShb, the SH2-Containing Adaptor Protein B of Schistosoma mansoni Regulates Venus Kinase Receptor Signaling Pathways
Source: PLoS One. 2016 Sep 16;11(9):e0163283. doi: 10.1371/journal.pone.0163283 (PMC5026347; doi:10.1371/journal.pone.0163283)

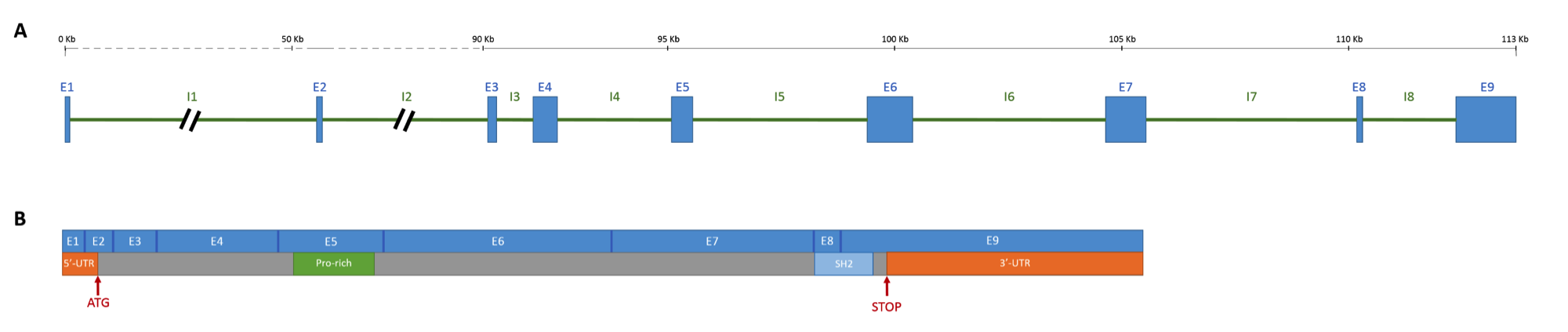

Supplement: S1 Fig — (A) Exon-intron structure of SmShb locus. Exons are shown as blue boxes and introns as green lines. Size scale is identical for introns and exons, except for I1 and I2 (larger than 30kb). (B) Schematic representation of the SmShb transcript indicating the coding sequence (in grey) with the Proline-rich domain in green and the SH2 domain in light blue. Untranslated regions (5’- and 3’-UTR) are indicated in orange. (TIF) [file pone.0163283.s001.tif]
