# Supplementary material for: SmShb, the SH2-Containing Adaptor Protein B of Schistosoma mansoni Regulates Venus Kinase Receptor Signaling Pathways
Source: PLoS One. 2016 Sep 16;11(9):e0163283. doi: 10.1371/journal.pone.0163283 (PMC5026347; doi:10.1371/journal.pone.0163283)
Supplement: S1 Table — (PDF) [file pone.0163283.s002.pdf]

Primer name	Sequence
Cl726Sh2protNt r	5'-GCCGCACCATCTGGTGGACATCTTGGT-3'
SmShbRaceNested5'r	5'-CGTAAAACAAAATGATCATCACCAATATCTGTTAGA-3'
Cl726Sh2protCt f	5'-GCTACCAGTACGTGGTACAACTCCGGT-3'
SmShbRaceNested3'f	5'-CAGATAATACTATTGGATCTCATGGACC-3'
SH2protFL f	5'-CCTGATAACTCTTTCCAATACAG-3'
SH2protFL r	5'-GTAAAAGTAATGGGGGAGAGGATCG-3'
SmShbFLRE f	5'-GGATCTCATGGACCTTTTGAATCGATATATGTCAACTCATACTC-3'
SmShbFLRE r	5'-GATTTATCTCGAGAAATGCAATACGACTGATTGGGAAGCGC-3'
SH2domain f	5'-GACATCAAAAATCAGAATTCTCACGTCATCATAGTAGTAGTAC-3'
SmShbdelSH2 f	5'-CAGCGATATCTTAACAACTTGAAAATCAACTATGGTTTCACCG-3'
SmShbdelSH2 r	5'-CGGTGAAACCATAGTTGATTTTCAAGTTGTTAAGATATCGCTG-3'
SmVKR1DK f	5'-GATGGGATTGTAAAAATTGGAGATaatgctCTTACTCGTG-3'
SmVKR1DK r	5'-CACGAGTAAGAGCATTATCTCCAATTTTTACAATCCCATC-3'
SmVKR1Y979F f	5'-CGTCAACCATTTGAAAACCTTTGTAATGAGCTAAAAGACATT-3'
SmVKR1Y979F r	5'-AATGTCTTTTAGCTCATTACAAAGGTTTTCAAATGGTTGACG-3'
SmVKR2F949Y f	5'-CGAACACGTAAACCTTATGAAGAACTATGCACAG-3'
SmVKR2F949Y r	5'-CTGTGCATAGTTCTTCATAAGGTTTACGTGTTCG-3'
SmShb-HIS1-f	5'-GATGGTGCGGCAGTTCATGAAATCTCTCC-3'
SmShb-HIS1-r	5'-GGTGGAGGTGGGAACCAAGTATTTGTATCGG-3'
SmShb RNAi T7 f	5'-TAATACGACTCACTATAGGGAGACCAACTTATCCTTCTGACAAG-3'
SmShb RNAi T7 r	5'-TAATACGACTCACTATAGGGAGAGAGTTGAAACTCTGTGTGTC-3'
LucT7 f	5'-TAATACGACTCACTATAGGGAGACTGGAGACATAGCTTACTG-3'
LucT7 r	5'-TAATACGACTCACTATAGGGAGAGGATCTCTCTGATTTTTCTTGCG-3'
SmShb qPCR f	5'-AGTCTTCTACACGATTCACTTT-3'
SmShb qPCR r	5'-TTAGTCGATTCTGAGGTTAGAT-3'
SmTubulin qPCR f	5'-AGCAGTTAAGCGTTGCAGAAATC-3'
SmTubulin qPCR r	5'-GACGAGGGTCACATTTCACCAT-3'
